# Evaluation of the quality of life of patients with chronic rhinosinusitis by means of the SNOT-22 questionnaire

**DOI:** 10.5935/1808-8694.20130010

**Published:** 2015-10-14

**Authors:** Pablo Pinillos Marambaia, Manuela Garcia Lima, Kleber Pimentel Santos, Amaury de Machado Gomes, Milena Magalhães de Sousa, Maria Eudiane de Macedo Marques

**Affiliations:** Otorhinolaryngologist certfed by the ABORL-CCF; Preceptor of the Otorhinolaryngology Department of the INOOA; PhD in Public Health - Federal University of Bahia - UFBA; Graduate program professor - Bahiana Medical School; MSc in Epidemiology - UFBA. Preceptor of the Otorhinolaryngology Department of the INOOA; MSc in Medicine - EBM. Preceptor of the Otorhinolaryngology Department of the INOOA; MD. ENT Intern - INOOA. EBMSP - Bahiana School of Medicine and Public Health. INOOA - Insttute of Otorhinolaryngology Associated ENTs

**Keywords:** indicators of quality of life, quality of life, sinusitis

## Abstract

SNOT-22 is a questionnaire used to assess the quality of life of patients with chronic rhinosinusitis (CRS). It is broadly utilized to assess the surgical treatment of patients with CRS. In Brazil there are no studies utilizing the SNOT-22 in non-surgical patients.

**Objective:**

To use the SNOT-22 questionnaire to assess the quality of life of individuals with chronic rhinosinusitis without previous surgery and with indication for clinical treatment.

**Method:**

Prospective and analytical cohort and cross-sectional controlled clinical trial. We had 2 groups, one made up of patients with CRS and another one with adult individuals without the sinonasal disease, consecutively seen in an otorhinolaryngology clinic in Salvador, Bahia, between August of 2011 and June of 2012. They all filled out the Consent Form, a registration form and the SNOT-22.

**Results:**

176 patients, 78 with CRS and 98 without the disease, the groups matched as far as gender, medication and respiratory allergies were concerned. Age was 40.7 + 13.5 years in the study group and 37.8 + 12.9 in controls (*p* = 0.26). The SNOT-22 median value in the study group was 53, compared to 8 in the control group (*p* = 0.001).

**Conclusion:**

Chronic rhinosinusitis reduces the quality of life of patients, according to the SNOT-22 questionnaire.

## INTRODUCTION

Chronic rhinosinusitis (CRS) is one of the most prevalent chronic diseases in the United States (USA) and Europe. It is estimated that the disease affects 31 million people per year in the USA^1^. CRS prevalence in the US is about 15% in the adult population, higher than arthritis and arterial hypertension^2^. Brazil still needs prevalence and incidence statistics associated with CRS; however, it is believed that the numbers are similar to those in US in pecentage^3^. Even being a highly prevalent disease, accurate data on epidemiology are few when compared to the large quantity of information on microbiology, diagnosis and treatment^4^.

The term chronic rhinosinusitis encompasses all inflammatory process, infectious or not, affecting the nasal cavity mucosa producing symptoms lasting for over 12 weeks^5^. Among the most common symptoms of this disease are nasal congestion or obstruction, hyposmia or anosmia, facial pain and anterior or posterior nasal secretion and facial pressure.

The CRS diagnosis is essentially clinical and straightforward when following the criteria established by the American Academy of Otorhinolaryngology, by the presence of two or more significant symptoms, such as: nasal obstruction/congestion/block, anterior or posterior rhinorrhea, hyposmia/anosmia, and facial pain/pressure, lasting for more than 12 weeks, besides nasal endoscopy and/or CT scan^6^.

The effects of chronic rhinosinusitis on the patient's quality of life (QL) and on productivity are well described on the world literature^7^. Comparing with patients without CRS, those with CRS report spending more days in bed, look for medical care more often, as well as alternative health-care professionals and mental health experts^8^. QL is a very important consideration in the assessment of the rhinosinusitis severity, on the clinical efficacy and quality of care of these patients^9^. These parameters are well established when utilized in surgical patients and on the assessment of the efficacy of this treatment; however, in Brazil, we still lack studies in outpatients with CRS without prior surgery and with indication of clinical treatment. It is logical that patients who are initially treated clinically may, after clinical treatment failure, be referred to surgery.

Numerous instruments have been developed in recent years to measure the QL in patients with chronic rhinosinusitis. In Brazil, such instruments were used from a simple translation into Portuguese and without proper validation^3^. Recently, the 20-Item SinuNasal Outcome (SNOT-20)^10^ and the 22-Item SinuNasal Outcome (SNOT-22)^11^ were validated for Brazilian Portuguese.

The goal of this study was to assess, by means of the SNOT-22 questionnaire, the quality of life of individuals with chronic rhinosinusitis without prior surgery and with an indication for clinical treatment.

## METHOD

A cross-sectional cohort, comparative, descriptive and analytical study was carried out, with a study group made up of patients with chronic rhinosinusitis and a control group made up of individuals without the sinonasal disease, all older than 18 years, consecutively seen in a reference Otorhinolaryngology clinic in Salvador - Bahia - Brazil between August of 2011 and June of 2012.

This study was approved by the Ethics Committee of the institution, under protocol #181/2011.

### Study Group

The study group was made up of patients with chronic rhinosinusitis. The diagnosis of chronic rhinosinusitis was defined using the criteria from the American Academy of Otorhinolaryngology, according to which the chronic rhinosinusitis is defined by the presence of two or more symptoms, such as: nasal obstruction/congestion/block, anterior or posterior rhinorrhea, hyposmia/anosmia, and facial pain/pressure, lasting for more than 12 weeks. We also used nasal endoscopy to look for purulent mucus secretion or middle meatus/ethmoid edema, and/or nasal cavity polyps or middle meatus^6^. Clinical criteria prevailed when the exam came back normal.

We took off the study the illiterate patients, smokers, and those with prior history of nasal surgery, patients with immunodeficiency, cystic fibrosis or primary ciliary dyskinesia, patients with benign or malignant nasal tumors, patients with granulomatous diseases and vasculitis, and those patients submitted to some type of treatment for CRS in the past 15 days before the deployment of the questionnaire, and those who refused to participate in the study.

### Control group

The control group was made up of patients without sinonasal disease. The inclusion criteria were: age higher than 18 years and no sinonasal disease defined on the negative response for the following questions: 1) Do you have some nasal problem?; Do you use or have you used any medication in the nose or for the nose? Illiterate, smokers and those who refused to participate in the study were taken off the study.

### Procedure

After selection, the patients were educated about the goals of the study and signed the Informed Consent Form. Following that, they filled out the SNOT-22 version validated for Brazilian Portuguese and a registration form with demographic data, besides the presence of comorbidities, respiratory and drug allergies.

### The Instrument

The 22-Item SinuNasal Outcome (SNOT-22) is a specific questionnaire to analyze quality of life in sinonasal diseases. It includes assessments of nasal, paranasal and psychological symptoms, and those associated with sleep. The SNOT-22^12^ is a questionnaire which is broadly used in the literature. It stems from the SNOT-20^13^^,^^14^ and primarily aims at assessing rhinosinusitis treatment. It has 22 questions about sinonasal symptoms and general status aspects, graded from zero to five; zero meaning no problems and five is the worst possible problem. The total sum of the questionnaire score, it numerically indicates the impact of the disease in the QL of the individual. It is considered the most adequate questionnaire to assess the quality of life of patients with chronic rhinosinusitis^15^.

### Statistical analysis

The results were plotted and analyzed in the SPSS-17 for Windows software.

Demographic data such as gender, schooling, comorbidities and allergies were exposed using the valid percentile. We used the chi-square test to compare the categorical variables among the groups. When the test assumptions were not met, we used the Fisher's test.

The SNOT-22 questionnaire score was described using the median and the interquartile interval because of the abnormal presentation of the results. We used the Mann-Whitney test to compare the median values of the total scores among the groups.

We considered an alpha error of *p* < 0.05 as significant.

## RESULTS

In this study we included 176 patients, 78 with chronic rhinosinusitis, in the study group and 98 from the control group. The groups matched as to gender and age (Table 1).

**Table 1:** Sociodemographic characteristics of the sample of patients with chronic rhinosinusitis (study group) and without sinonasal disease (control group).

Variables	Study Group	Control Group	Significance
	(n = 78)	(n = 98)	(P)
Gender (%)			
Male	32 (41)	40 (40.8)	1.0
Female	46 (59)	58 (59.2)	1.0
Age	40.7 + 13.5	37.8 + 12.9	0.26
Schooling (%)			
1^st^ degree	2 (2.5)	13 (13.3)	0.01*
2^nd^ degree	21 (26.6)	29 (29.6)	0.66
3^rd^ degree	50 (63.3)	52 (53.1)	0.17
No information	6 (7.6)	4 (4.1)	0.31

Study Group: Chronic rhinosinusitis; Control Group: Patients without sinonasal disease. * Level of significance = *p* < 0.05.

The mean age was 40.7 ± 13.5 years in the study group and 37.8 ± 12.9 in the control group (p = 0.26).

Comorbidities, medication and respiratory allergies are shown on Table 2.

**Table 2:** Comorbidities and allergies to medication and respiratory allergy.

Variables	Study Group (n = 78)	Control Group (n = 98)	Significance (p)
Comorbidities			
SAH	10	10	0.81
DM	6	3	0.307
Asthma	7	1	0.026*
Hypothyroidism	2	1	0.6
Drug allergy (%)			
Yes	18 (22.8)	13 (13.3)	0.112
No	60 (77.2)	85 (86.7)	–
Respiratory allergy (%)			
Yes	5 (6.3)	7 (7.1)	1.0
No	73 (93.7)	92 (92.9)	–

Study group: Chronic rhinosinusitis; Control Group: Patients without sinonasal disease. SAH: Systemic Arterial Hypertension; DM: Diabetes Mellitus. * Level of significance *p* < 0.05.

Schooling level was analyzed and there was a difference between the percentage of individuals with only junior high school education among the groups, and in the control group there were more individuals with this characteristic (p = 0.01).

A comparison of the median values in the total score of the SNOT-22 among the groups are depicted on Table 3.

**Table 3:** SNOT-22 Quality of Life Score - Median/IC.

Variable	Study Group	Control Group	Significance (p)
SNOT-22	53 (35)	8 (10)	0.001*

SNOT-22: 22-Item SinuNasal Outcome Test. * Level of significance *p* < 0.05. Mann-Whitney U test.

**Graph 1:**
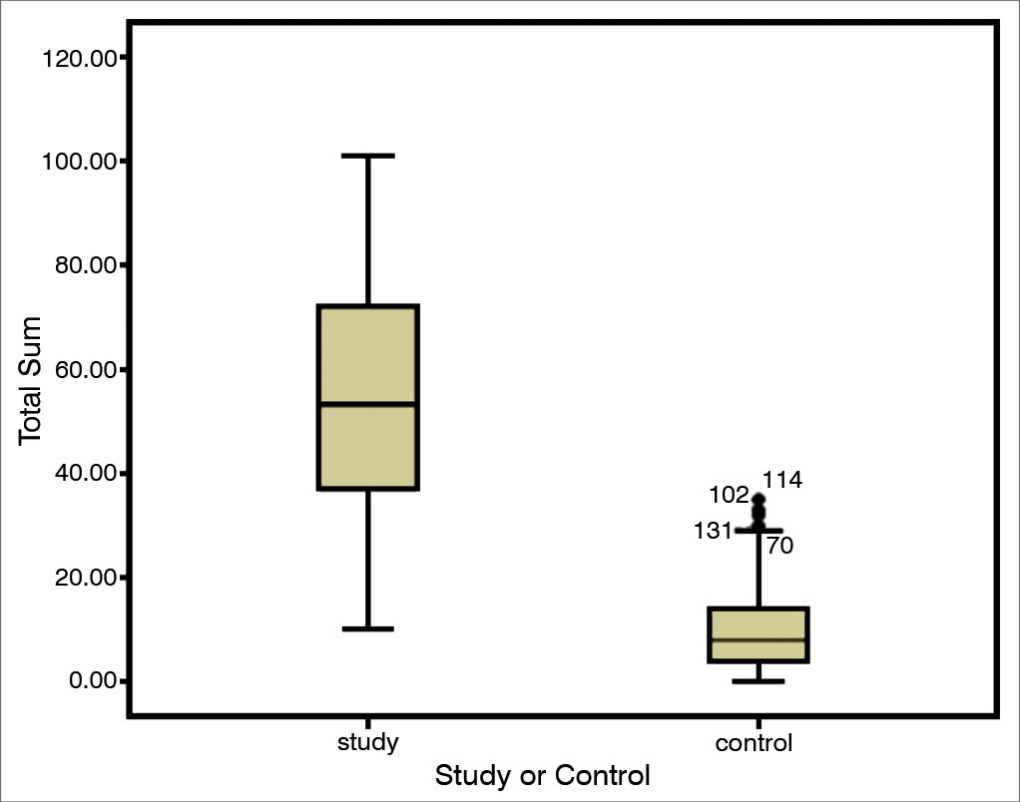
Comparison of the quality of life of sick people and the normal population. Comparing medians. Study group: Chronic rhinosinusitis; Control Group: Patient without the disease; Mann-Whitney test. *p* = 0.001.

Graph 1 shows the comparison of the median values from the SNOT-22 total score between the two groups.

## DISCUSSION

We found a marked difference between the patients' scores with chronic rhinosinusitis and the patients without this condition, which confirms the high impact of chronic rhinosinusitis in the quality of life of these patients.

This data is not a novelty in the world literature, but it is the first time in Brazil that we have data on an outpatient population without prior surgery and with indication of clinical treatment. Even in outpatients, CRS has a sensible negative impact on the QL.

We must stress that this sample is made up of patients whom, at the time of inclusion, did not have surgical indication; however, some of them, after not responding to clinical treatment, were referred to surgery and were treated.

In the study carried out to validate the SNOT-22 for the Portuguese language, Kosugi et al.^11^ employed the questionnaire in 89 patients before and after the sinonasal surgery, obtaining a mean preoperative score of 62.39 in the group with the disease, compared to 53.8 of our sample of outpatients. Hopkins et al.^12^, who were the first to validate this questionnaire in the United Kingdom, employed the questionnaire in 2,077 surgical patients and obtained a pre-operative score of 41.7. This difference between the Brazilian studies and the English study, can be associated with the difference in life and culture between the two nations. As to the national reality, the difference may correspond to the fact that our sample stems from a clinic which sees patients from health plans and private patients, who have a socio-economical level, likely better than that of patients from the Kosugi et al.^11^ sample. Another possible explanation is that the patients utilized in our study were all outpatients who were clinically treated, in other words, without surgical indication at the time of the study, and the Kosugi patients all had surgical indication, presumably with the worst scores.

The control group had a score of 08. As with the population assessed by Gillett et al.^16^, in the present sample, the patients were not free from symptoms by the SNOT-22. We believe that this is primarily due to the fact that the questionnaire had general health domains such as “fatigue” or “difficulty to sleep”, which may be associated with other non-reported or not-investigated medical conditions and, at last, in way the healthy ones were selected, only by the response of the subjects, which may have included possible bearers of CRS who did not have their formal diagnosis.

Our study, just like the international literature^9^^,^^17^, collected the data by means of the self-application of the instrument, in order to remove the interviewer bias. For that, it was necessary, as an inclusion criterion to consider the individuals' education. We are fully aware that, since we live in a country with striking social inequalities, taking illiterate individuals off, may not represent the general reality of our country, nonetheless, our sample closely represents a part of the educated population who has health plan coverage, and such group is growing in recent years with improvements in the social conditions of the population^18^.

The difference between the groups investigated as to schooling was not important, since after stating that the control group had a higher number of individuals completing junior high school only, we compared the median values from the “junior high school complete” group with the other subjects in the sample, which showed no statistical difference between them.

One significant difference between the groups was that the sick individuals were more frequently affected by asthma. Such finding corroborates a well settled and broadly accepted information, which is the association between asthma and CRS.

Our sample was unable to accurately analyze the respiratory allergies, given that this information was reported by the patient in a categorical fashion, only as respiratory allergy “present” or “absent” and may not have been properly understood by the individuals. Another possibility is that those with allergic rhinitis, who presumably belonged to the study group may not have their allergy documented by laboratorial exam. By the same token, as per previously stated, the inclusion criteria of the control group may have selected patients with sinonasal disease who were not formerly diagnosed.

The advantages of this study are the fact that it was the first so far to use the SNOT-22 instrument validated to Portuguese, the first to use it in a population without prior surgery and with indication of clinical treatment, its use in a population covered by health plans and the fact that it uses the same international methodology of self-application, to which the instrument was created. As for limitation, we mention the non-characterization of the presence of allergies, as well as the fact that we excluded the illiterate, a significant portion of our population.

## CONCLUSION

The answers we had from the SNOT-22 questionnaire show worse quality-of-life scores from patients with chronic rhinosinusitis without previous surgery, referred to clinical treatment, when compared to the group without sinonasal disease.
